# Whole genome resequencing of the Iranian native dogs and wolves to unravel variome during dog domestication

**DOI:** 10.1186/s12864-020-6619-8

**Published:** 2020-03-04

**Authors:** Zeinab Amiri Ghanatsaman, Guo-Dong Wang, Hojjat Asadollahpour Nanaei, Masood Asadi Fozi, Min-Sheng Peng, Ali Esmailizadeh, Ya-Ping Zhang

**Affiliations:** 1 Department of Animal Science, Faculty of Agriculture, Shahid Bahonar University of Kerman, PB 76169-133, Kerman, Iran; 2 Yong Researchers Society, Shahid Bahonar University of Kerman, PB 76169-133, Kerman, Iran; 3 State Key Laboratory of Genetic Resources and Evolution, Kunming Institute of Zoology, Chinese Academy of Sciences, No. 32 Jiaochang Donglu, Kunming, 650223 Yunnan China; 4 State Key Laboratory for Conservation and Utilization of Bio-Resources in Yunnan, Yunnan University, Kunming, 650091 China

**Keywords:** Single nucleotide variant, Copy number variant, Structural variant, Fertile crescent

## Abstract

**Background:**

Advances in genome technology have simplified a new comprehension of the genetic and historical processes crucial to rapid phenotypic evolution under domestication. To get new insight into the genetic basis of the dog domestication process, we conducted whole-genome sequence analysis of three wolves and three dogs from Iran which covers the eastern part of the Fertile Crescent located in Southwest Asia where the independent domestication of most of the plants and animals has been documented and also high haplotype sharing between wolves and dog breeds has been reported.

**Results:**

Higher diversity was found within the wolf genome compared with the dog genome. A total number of 12.45 million SNPs were detected in all individuals (10.45 and 7.82 million SNPs were identified for all the studied wolves and dogs, respectively) and a total number of 3.49 million small Indels were detected in all individuals (3.11 and 2.24 million small Indels were identified for all the studied wolves and dogs, respectively). A total of 10,571 copy number variation regions (CNVRs) were detected across the 6 individual genomes, covering 154.65 Mb, or 6.41%, of the reference genome (canFam3.1). Further analysis showed that the distribution of deleterious variants in the dog genome is higher than the wolf genome. Also, genomic annotation results from intron and intergenic regions showed that the proportion of variations in the wolf genome is higher than that in the dog genome, while the proportion of the coding sequences and 3′-UTR in the dog genome is higher than that in the wolf genome. The genes related to the olfactory and immune systems were enriched in the set of the structural variants (SVs) identified in this work.

**Conclusions:**

Our results showed more deleterious mutations and coding sequence variants in the domestic dog genome than those in wolf genome. By providing the first Iranian dog and wolf variome map, our findings contribute to understanding the genetic architecture of the dog domestication.

## Background

The dog (*Canis familiaris*) was likely the first domesticated animal and the only one humans’ friend in the past [21, 71]. Genetic studies and archaeological discoveries showed that the dogs have a common ancestor with the gray wolf (*Canis lupus*) [22, 68, 73]. In the Southwest Asia, major–scale farming extended within the so-named Fertile Crescent (FC) where the independent domestication of plants and animals had led to shifting from gathering and hunting to sedentary farming following expansion of the first complex societies [23, 78]. Mostly, agricultural developments happened in the eastern horn of FC especially Elam (covering a region of southern Iraq and Iran), joining Mesopotamia and Iranian plateau [5]. Dogs are often drawn in art at ancient times in several parts of Southwest Asia [21, 55]. Therefore, one of the most theories about the geographical origin of the domestic dog has been that they originated in Southwest Asia, presumably in the FC [21]. In addition, the Middle East has been proposed as the beginning of domestic dog for great haplotype sharing between wolves and dog breeds [69] although this hypothesis has been questioned due to dog-wolf introgression [7, 8, 30] rather than an indication of Middle Eastern origins. The dog is a notable instance of variation under domestication, however the evolutionary processes underlying the genesis of this diversity are weakly realized.

In recent years, advance in high-capacity genome examining techniques*,* especially whole genome sequencing, SNP genotyping array and comparative genomic hybridization (CGH) arrays have authorized the recognition of genome-wide structural variants. The array methods have limited resolution and low sensitivity because their performance is strongly depending on the marker frequency and particularly constructed non polymorphic markers,[6, 45, 57] thus they cannot detect small copy number variations (CNVs) (< 10 kb) and cannot precisely identify boundaries of CNVs [77]. Next-generation sequencing methods provide a high-accuracy base-by-base vision of the genome and capture all variants by different size that might otherwise be missed, and all these are important and have significant effects on an extensive range of traits in domesticated animals. For examples: Fear and anxiety will be increased by increasing of expression of *GRIK2* gene in domesticated species than their wild species including rabbit, guinea pig, dog and chicken [42], *MC1R* gene makes coat color variants in pig [28] and mutation in *TSHR* gene influences seasonal reproduction in chicken [60].

CNVs can also have major impact on phenotypic variation in humans, animals and plants. For example, previous studies have found CNVs that are involved in traits related to pea-comb and late feathering in chicken [27, 74], polledness in goat [53], hair ridge in dog [35], health and production in cattle [13] and adaptability in dog [10, 72]. In this work for the first time, we sequenced the whole genomes of 6 canids from the same geographical range (three Iranian wolves and three Iranian dogs) with an average depth of 16X. One of the sequenced dogs, Qahderijani, is a mastiff ecotype dog originating in Qahderijan, Iran, which is located in FC belt (surrounding areas of FC). Other two sequenced samples were collected from the Saluki, a hunting dog breed, which is belonged to the FC region. Saluki is also considered as one of the long-marathon runner dog breeds in the world, as its incredible endurance enables it to run for several miles.

In our analysis of the Iranian dog and wolf sequences, we applied assembly version canFam3.1 as a reference sequence [43]. SNPs and small Indels were called in this research as differences between the recently gained genome sequences and reference sequence. We identified a total number of 12.45 and 3.48 million SNPs and small Indels, respectively. Valid algorithms were applied to analyze 6 genomes to get highly reliable CNVs and SVs. The potentially breed-specific CNVRs were defined and the functional relation of the SV and CNVR-covering genes was further evaluated by GO enrichment analysis. Genome-wide analysis indicates more genetic diversity in the dog genome than that in the wolf genome. The genomic annotation results from different variation types proposed increasing the percentage of genomic variations in the coding and the regulatory regions than that in the intron and intergenic regions during domestication, which is substantial contributor to the currently detected differences between dog and wolf. Also, our genomic comparison results between dog and wolf showed that genes engaged in neurological, digestion and metabolism processes had a considerable effect on the progress of dog domestication. The CNVs reported in this research are enriched for olfactory and immune system genes.

## Results

### Sequencing output

Illumina Paired-end sequencing was performed for all 6 individuals (Additional file 1: Table S1 and Fig. S1). After filtering, the range of total high-quality sequence data was from 42.1 Gb (Sample ID: #GW1) to 51 Gb (#DogQI), and the coverage varied from 14.51 x (#GW1) to 17.15 x (#GW2) (Additional file 1: Table S2). The range of mean insert sizes and their standard deviations in sequenced data for all samples was from 280.06 to 331.86 and from 27.12 to 33.94, respectively. Using the paired-end DNA sequencing reads together with a uniform read length (by a length of 125 bp) (Additional file 1: Table S1), we called all Indels [49, 65]. We also used uniform depth of coverage across individual genomes for increasing reliability of CNV calling (Additional file 1: Table S2).

### SNP detection and annotation

The SNPs were detected through aligning sequences to the reference genome. A total of 12.45 million SNPs were detected in all individuals (10.45 and 7.82 million SNPs were identified for all studied wolves and dogs, respectively) (Additional file 1: Table S3 and Fig. S2).

**Fig. 1:**
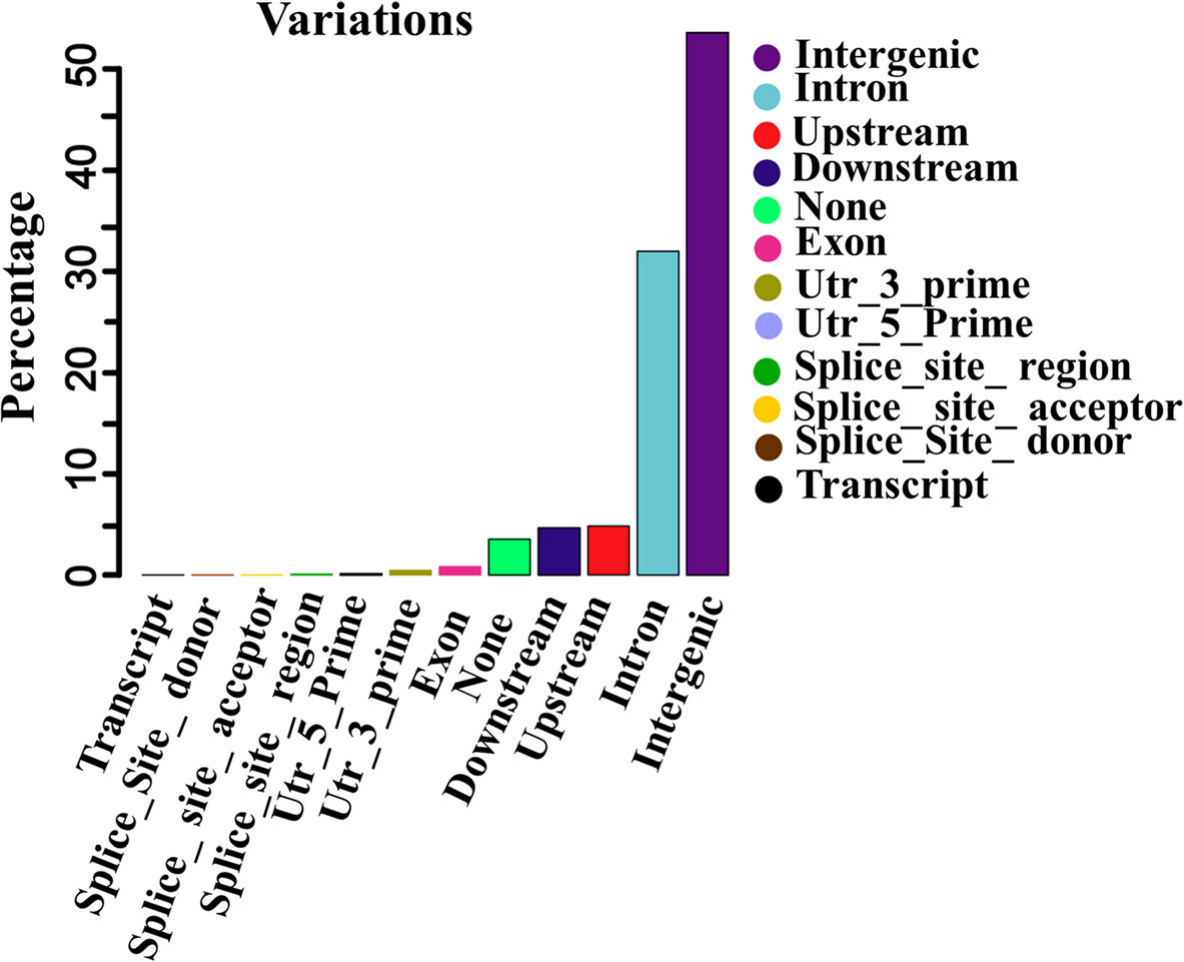
The proportion of SNPs present in each genomic regions, including intergenic, introns, exon, transcript, upstream, downstream, three prime untranslated regions (3′*-*UTR) and five prime untranslated

We also obtained the ratio of transitions to transversions (Ti/Tv) for all heterozygous and homozygous SNPs identified across the 6 individual genomes. The number of heterozygous SNPs was higher than homozygous SNPs. The Ti/Tv ratio varied from 1.99 (#DogQI) to 2.07 (#GW3) (Additional file 1: Table S4) in all SNPs. Figure 1 illustrates the proportion of SNPs present in each genomic regions, including intergenic, introns, exon, transcript, upstream, downstream, 3′ untranslated regions (3′*-*UTR) and 5′ untranslated regions (5′*-*UTR). Our results indicate that most of the SNPs are located in the intergenic (53.57%) and intron (31.99%) regions (Additional file 1: Table S5). The total number of synonymous SNPs (silent SNPs, 68,899) were more than the total number of non-synonymous SNPs (nonsense and missense SNPs, 46,789) (Additional file 1: Table S6). Also, our genomic annotation results showed that the proportion of wolf SNPs in intron (31.85 vs 31.81) and intergenic (53.92 vs 53.52) regions, and in exon (0.81 vs 0.84) and 3′-UTR (0.43 vs 0.46) regions, was higher and lower, respectively, than that in dog genome.

### Small Indels detection, annotation and gene ontology

Indels were detected using aligning sequences to the reference genome. The number of Indels was calculated for all individuals (Additional file 1: Table S3). A total number of 3.48 million Indels were detected across the 6 individual genomes, 2.24 million and 3.11 million of which were for 3 dogs and 3 wolves, respectively. We also calculated the number of heterozygous and homozygous Indels across individual genomes (Additional file 1: Table S4). The proportion of heterozygous Indels (52.12) was higher than the proportion of homozygous Indels (47.59) for all individuals. The total number of small insertions and small deletions across all the 6 canid genomes were 1.58 and 1.9 million, respectively (Additional file 1: Table S7). We drew the Indel length histogram for 3 dogs (Additional file 1: Fig. S3), 3 wolves (Additional file 1: Fig. S4) and across all individual genomes (Additional file 1: Fig. S5). The results showed that the Indels of 1 bp in length across the 6 individual genomes had the highest frequency and the deletions of the same size were more frequent than the insertions. According to our annotation results (Additional file 1: Table S8), most of the Indels are located in intergenic (22,832,990, 53.79%) and intron regions (1,476,727, 34.45%), and after that in upstream (235,329, 5.54%), downstream (210,059, 4.95%), exon (10,407, 0.25%), 3′-UTR (19,671, 0.46%), 5′-UTR (5483, 0.14%), and transcript (103, 0.002%) regions. The percentage of small Indels that are located in upstream, 5′-UTR, 3′-UTR, exon and transcript regions across 3 dog genomes was higher than that across 3 wolf genomes, but the percentage of Indels that are located in downstream, introns and intergenic regions across 3 wolf genomes was higher than that across 3 dog genomes. We obtained 21,104 genes from ensemble, through the annotation of a total of 3.48 million small Indels. We then performed gene ontology (GO) and Kyoto Encyclopedia of Genes and Genomes (KEGG) pathway analysis for all detected genes (Additional file 1: Table S9 and Table 1). GO analysis categorized genes related to small Indels in the three main classes (molecular function, biological process and cellular component) (Additional file 1: Table S9). The KEGG pathway analysis for all detected small Indels showed that two pathways related to cancer and Melanoma (usually but not always, a cancer of the skin) were enriched in both dog and wolf genomes (Table 1).

**Table 1:** KEGG**_** pathways enriched among different types of variants

Type of variants	KEGG_ *pathways* ID	Description	Animal	*P*-value (wolf)	*P*-value (dog)
Small indels	hsa05200	Pathways in cancer	Both	0.0020	0.0010
Small indels	hsa05218	Melanoma	Both	0.0487	0.0405
translocation	hsa04740	Olfactory transduction	Both	0.0005	0.0016
Structural variant (translocation)	hsa04612	Antigen processing and presentation	Both	0.0004	0.0385
Structural variant (translocation)	hsa01200	Carbon metabolism	Dog	–	0.0996
Structural variant (inversion)	hsa04973	Carbohydrate digestion and absorption	Dog	–	0.0613
Structural variant (inversion)	hsa04970	Salivary secretion	Dog	–	0.0804
Structural variant (indels)	hsa04662:	B cell receptor signaling pathway	Both	0.0085	0.0165
Structural variant (indels)	hsa04660:	T cell receptor signaling pathway	Both	0.0163	0.0655
CNV	hsa04740	Olfactory transduction		4.04E-15	0.0957
CNV	hsa04260	Cardiac muscle contraction	Dog^a^	–	0.0667
CNV	hsa00500	Starch and sucrose metabolism	Dog		0.0119
CNV	hsa04020	Calcium signaling pathway	Both	0.0775	0.0238
CNV	hsa00140	Steroid hormone biosynthesis	Both	0.0385	0.0802

^a^Only enriched in the Saluki dog breed

### SVs detection, annotation and gene ontology

In this study, we obtained genomic SVs including insertions, deletions, tandem duplication, translocations (inter and intra chromosomal) and inversions from three dogs and three wolves (Additional file 1: Table S10; Additional file 2: Table S16, Additional file 3: Table S17 and Additional file 4**:** Table S18). To investigate the potential functional roles of all different SVs types, all genes that were completely or partially overlapped with genomic regions including, Indels (insertion and deletion), inventions and complex SVs (inter and intra chromosomal translocations) were retrieved from Ensemble (Additional file 1: Table S11). Annotation results from SVs showed that in general the percentage of coding sequences variants in dog genome is higher than that in wolf genome (Additional file 1: Figs. S6-S13). Also, gene set enrichment analysis showed three enriched categories related to “covering molecular function”, “biological process” and “cellular component” (Additional file 1: Table S12). The most conspicuous cluster terms related to dog and wolf individuals were “cellular carbohydrate metabolic process (*P*-value, 0.04)” and “nervous system development (*P*-value, 0.03)”, respectively. We also identified some candidate genes associated with olfactory and immune systems (Additional file 1: Table S12 and Table 1).

### CNV detection

**Fig. 2:**
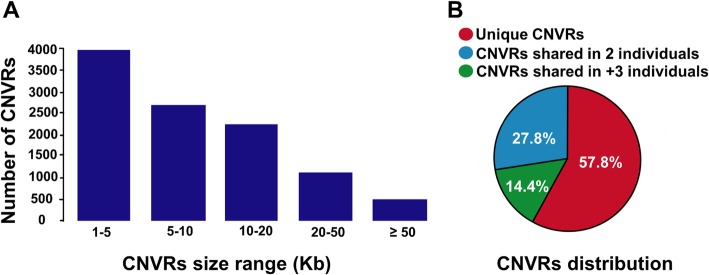
The length and distribution of CNVRs. **a** a total of 6702 (63.39%) and 494 (4.67%) out of all CNVRs had sizes ranging from 1.049 to 10 kb and longer than 50 kb in size, respectively. **b** 4466 (42.25%) CNVRs are shared in at least two individuals and 6105 (57.75%) CNVRs present in only one individual

We obtained putative CNVs for all individuals using CNVnator program and the mean number of CNVs per individual was 4143.83, ranging from 2871 to 5437 (Additional file 1: Table S13). For all of the autosomal CNVs categorized as gain, the mean copy number value of six individuals was 3.57 and the maximum copy number assessment was 174.472 on chromosome 7 (chr7) of wolf. The results showed that the number of gains in the three dog genomes was higher than those in the three wolf genomes (Additional file 1: Table S13). A total of 10,571 CNVRs were obtained from overlapping of all CNVs across the 6 individuals (Additional file 5: Table S19), including 1–38 and X chromosomes, ranging in size from 1.05 kb to 3433.35 kb with an average of 14.63 kb and a median of 7.05 kb, covering 154.65 Mb, or 6.41%, of the assayed canFam3.1 genome (Table 2). CNVRs were divided into three groups, including 6400 loss, 3916 gain and 255 both (gain and loss) events (Additional file 5: Table S19). Deletion:duplication ratio in the total CNVRs was 1.96. Among all CNVRs, 6105 (57.75%) were found in a single individuals (singleton), 1522 (14.39%) shared in two individuals, and 2944 (27.84%) shared in at least three individuals (Fig. 2b). A number of 6702 (63.4%) CNVR events were less than 10 Kb while 494 (4.7%) of the CNVRs were longer than 50 kb in size (Table 2 and Fig. 2a). The highest and lowest numbers of CNVRs belonged to chromosomes 18 and 35, respectively (Additional file 1: Fig. S14 and Additional file 6: Table S20).

**Table 2:** Size distribution of the CNVRs detected by CNVnator

Summary statistic of CNVRs	Gain	Loss	Both (loss and gain)	Total
Number of CNVRs	3916	6400	255	10,571
Total length (Mb)	83.75	47.28	23.62	154.65
Mean length (Kb)	21.39	7.39	92.62	14.63
Median length (Kb)	11.70	4.49	38.99	7.05
1 ≥ Kb to < 5 Kb	555 (14.17%)	60 (0.94%)	–	3996 (37.80%)
5 ≥ Kb to < 10 Kb	1119 (28.57%)	3441(53.76%)	14 (5.49%)	2706 (25.59%)
10 ≥ Kb to < 20 Kb	1160 (29.62%)	1573 (24.57%)	45 (17.64%)	2252 (21.30%)
20 ≥ Kb to < 50 Kb	750 (19.15%)	1047 (16.35%)	189 (74.11%)	1123 (10.62%)
50 ≥ Kb	332 (8.47%)	279 (4.35%)	7 (2.74%)	494 (4.67%)

### CNV annotation and gene ontology analysis

The annotation of results from CNVs showed that the percentage of CNVs in coding sequences (14% vs. 6%) and 3′-UTR (6% vs 0) region in the dog genome was greatly higher than that in the wolf genome, but the percentage of CNVs in the intergenic regions (22% vs. 14%) in wolf genome was greatly higher than that in the dog genome (Additional file 1: Figs. S15 and S16). To achieve potential functional roles related to the putative CNVs, all genes that completely or partially overlapped with these CNVs were detected from Ensemble. A total of 8595 genes were retrieved, including 6703 of the CNVs. Results of GO analysis showed that some general genes associated with olfactory and immune systems are enriched among the CNV gains in dog and wolf (Additional file 1: Table S14). All the terms related to olfactory system are over-represented (*P*-value <0.01) in the wolf compared with those in the dog (Additional file 1: Table S14 and Table 1). The term “Starch and sucrose metabolism (*P*-value, 0.01)” is enriched in the dog CNV gains (Table 1). Also, our result showed that some categories including “cardiac conduction (*P*-value, 0.03)”, “actin filament (*P*-value, 0.037)”, “muscle filament sliding (*P*-value, 0.02)”, “ATP binding (*P*-value, 3.46E-04)” and “calcium ion binding (P-value, 0.001)” are enriched among the CNV gains in the Saluki breed (Additional file 1: Table S14).

### Comparison with previous dog CNV studies

To compare the identified CNVRs in this work with those previously published studies, all CNVR coordinates from canFam2 were migrated to canFam3 using the UCSC leftover program. In our results, 4454 CNVRs (42.1%) were overlapped by four previous studies, and the remaining 6117 (57.865%) were considered as novel CNVRs (Additional file 1: Table S15 and Additional file 7: Table S21).

### Visualization of structural genomic variation

**Fig. 3:**
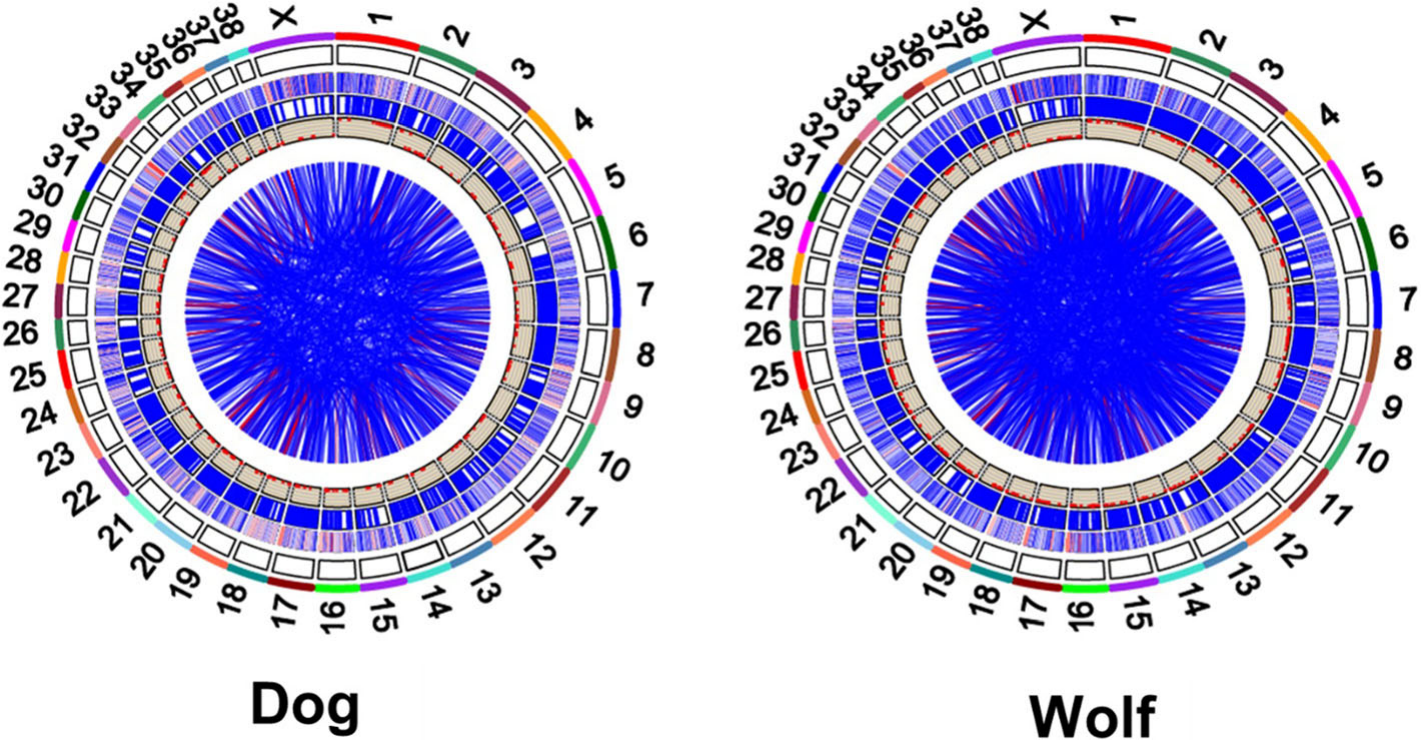
Graphical visualization of predicted SVs for dog and wolf. Starting from outside of the circle, the following features are shown: chromosome ideograms, heatmap plot of copy number variation with color according to the CNV value computed by CNVnator, genomic locations of tandem duplications, genomic locations of inversions and genomic locations of intra and inter- chromosomal links

For visualizing similarities and differences of positional relationships and genome structure between dog and wolf genomes, we drew maps of circular genomes for dog and wolf (Fig. 3).

### Identification of deleterious mutations

**Fig. 4:**
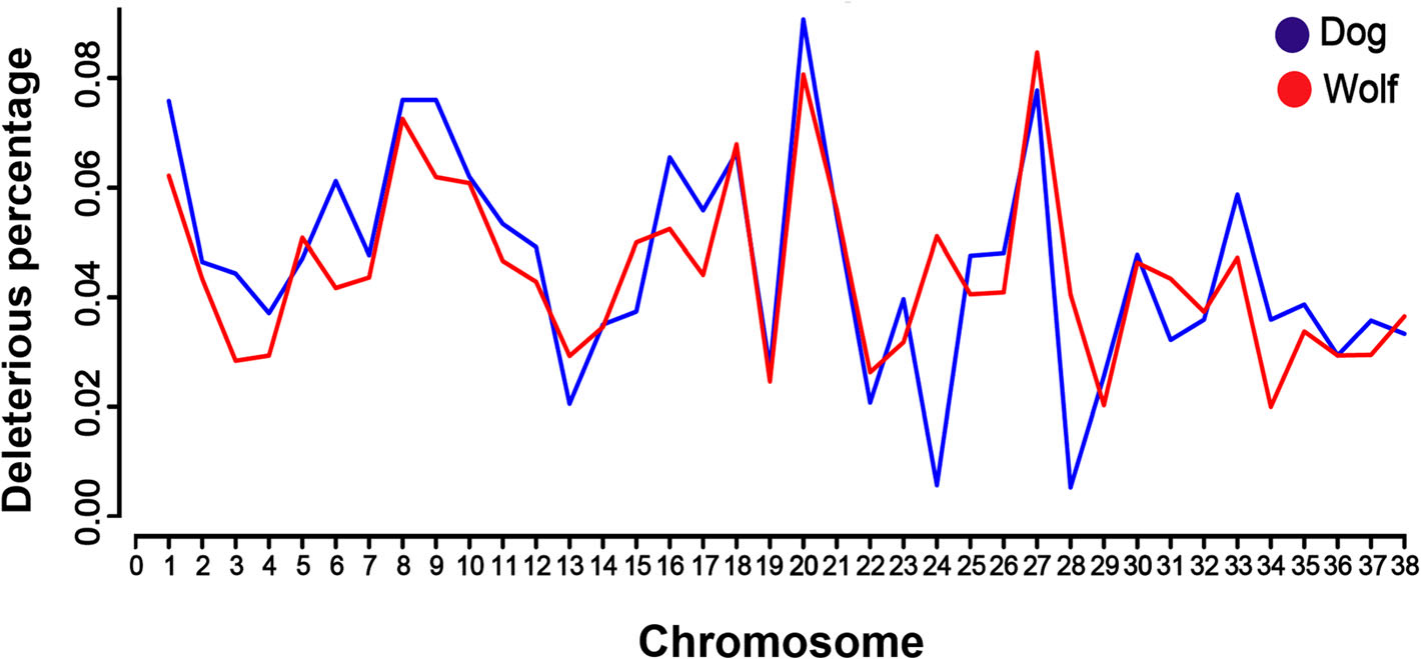
The proportion of deleterious mutations in wolf and dog chromosomes

Population genetic processes due to reduced population size, such as inbreeding depression and bottlenecks, have a profound impact on the genetic makeup of a species including levels of deleterious variation [16, 37, 39]. Our results indicated that the proportion of deleterious mutations varied between wolf and dog chromosomes (Fig. 4), and more deleterious mutations are in dog genome, compared with their wild ancestor.

## Discussion

Analysis of high-quality next-generation sequencing data clearly showed the difference of the distribution and impact of the genomic variations between dog and wolf. We calculated ti/tv ratio for all individuals (1.99 to 2.07) (Supplementary Table S4) that is an indicator of false positive ratio for SNP calling steps [11, 33]. Our finding revealed the high precision of the identification of single-nucleotide mutations in this research. In addition, the results of this research, similar to previous study [62], showed that most of the SNPs are located within introns or between genes, and the number of synonymous SNPs was higher than non-synonymous SNPs. The majority of small Indels (95.89% in dog and 95.64. % in wolf) were less than 10 bp in length, similar results were reported in a study of Indels in chicken [76]. Two cancer and melanoma pathways were enriched with small Indels in both dog and wolf. The previous studies showed that cancer and melanoma diseases were created by genomic variants especially small Indels in both dogs and human [31, 34, 70]. Our results highlighted the importance of dogs as a model for surveying human diseases.

We detected 10,571 CNVRs with a mean of 4143.83 CNVs per sample in the canine genome. In our results, similar to those reported in dog and wolf [19, 47, 51, 52], human [24, 59] and mouse [32], loss events were more prevalent than gain events (1.63 fold). This may mirror the greater relative hardness of identifying gains because of the smaller relative alteration in copy number (3,2 versus 2,1). Loss events included shorter genomic sequences than gains on median (4.499 kb vs. 11.699 kb), mean (7.387625 kb vs. 21.38724 kb) and total (47.280800 Mb vs. 83.752434 Mb) (Table 2). This could show that duplications are less likely to be cleaned by purifying selection [6]. A total of 4466 (42.25%) CNVRs are seen in at least two individuals and 6105 (57.75%) CNVRs present in only one individual. Percentage of singletons obtained in this work is in agreement with that reported in previous studies related to CNV studies in human [59], dog [51] and chicken [77]. We realized that the CNVRs were non-randomly distributed across the canid genome (Table S20). Chromosome 32, for example, has 2.03% of sequences displaying copy number variable, whereas chromosome 18 has 42.79% of sequences with copy number variation (Supplementary Table S20). In general, the chromosomes 9 (13.03%), 26 (14.97%) and 18 (42.78%) showed a high percentage of the CNVRs.

The terms “sensory perception of smell”, “detection of chemical stimulus” and “Olfactory transduction” were enriched among the CNV gain regions in dog and wolf (over-represented, *P* < 0.01), which are involved in sensory perception. Both wolf and dog develop olfaction, audition and vision by 2 weeks, 4 weeks and 6 weeks of age on average, respectively [44]. Wolf pups start to investigate their environment at 2 weeks of age while they are blind and deaf, and must depend mainly on sense of smell, while dog pups start to investigate their environment at 4 weeks of age [44]. In a previous study, the fraction of olfactory receptor pseudogenes in dog and wolf was 17.78 and 12.08%, respectively, however, difference between these values in dog and wolf was not significant [80]. In one another study, no difference in the olfactory capacity of the dog breeds, which have been chosen for their smelling ability and the hand-breaded grey wolves, was reported [54]. However, our results suggest an importance rule for olfaction during dog domestication. Six of the GO terms belonged to CNV gain regions in this study are also similar to those that were presented using aCGH method in dog [12].

GO term enrichment analysis showed that gene families involved in sense of smell and immune system commonly rapid growing for their importance rule in the organism terms answering to fast changes in the environment and fitness, also they have been frequently identified in CNV regions of multiple mammalian genomes [2, 75, 82]. Go terms related to heart and muscle functions such as “cardiac conduction” and “actin filament” were only enriched in the CNV gain regions in Saluki dog breed. These results can be expected because Saluki is a hunting dog breed which is considered as the long marathon runner of the canine world and its incredible endurance enables the dog to run for many miles [4, 48]. It has been presented that endurance exercise training makes a number of cardiac adaptations to marathon running [63]. Also in dog, a more recent study has reported specific CNVs related to hunting in the BRA breed [26]. A fundamental number of CNVs (~ 42%) from this work are compatible with those identified in previous studies in dogs and wolves. In addition, a substantial number of detected enriched Go terms of this study (~ 31%) are concordant with previous research in dogs and wolves [12]. This compatibility with the previous studies, in conjunction with the identification of the CNVs specific to the Saluki breed, lends more support to the CNVs identified in this work. The difference between the CNVs detected in the study herein and those described previously can be related to the particular breeds studied and also the difference between the methods used. Generally, the CNVs that are identified by read-depth analysis are on average much smaller than those detected by aCGH.

The total numbers of SNPs (10.45 million vs 7.82 million), Indels (3.11 million vs 2.24 million), deletions (18,628 vs 13,059), inversions (401 vs 334), inter (706 vs 520) and intra (421 vs 359) chromosomal translocations regions were higher in the wolf genome than those in the dog genome, while the total number of CNVs located at gain (2277 vs 521) and insertions (352 vs 311) regions in the dog genome were higher than those in the wolf genome. It has been accepted that gene duplication through yielding material for selection, mutation and drift can be a chief source of recentness in evolution [81].

Our results from genome analysis for dog and wolf revealed reduction of the genomic diversity during dog domestication. A population bottleneck occurred in the wolves thousand years ago after a population expansion occurred by human through artificial selection on specific traits leading to different breeds of dogs [3, 30]. The effective population size in wolves is higher than that in dogs so higher genome diversity in wolves is expected compared to dogs [3, 30]. Our results from two components of genetic variation sources including SVs and CNVs confirmed that the novel adaptations permitted the primal ancestors of recent dogs to live on a diet with high starch compared to the carnivorous diet of wolves, which is an essential step in the primal domestication of dogs [9, 10, 30, 64, 73]. The term “nervous system development” was enriched among SVs in wolf and is indicative of reducing aggression in the first steps of animal domestication [70]. This term is defined as a process that particular result is the development of nervous tissue over time from its production to its developed shape.

Annotation results from different types of genomic variations showed that in general the percentage of genomic variations in intron and intergenic regions in wolf genome is higher than that in dog genome while in coding sequences and 3′-UTR in dog genome is higher than that in wolf genome.

It seems that domestication and its related processes such as relaxed selection have an important role in increasing the percentage of genomic variation in the coding and the regulatory sequences of dog genome. The relaxation of selection likely increases the functional genetic diversity throughout the genome of the dog and this diversity includes both the genes and the elements involved in gene expression [14, 25]. Previous studies have shown that the extensive selection for phenotypic and behavioral traits, have resulted in morphological diversity within the domestic dog [67]. Also, it has been suggested that after domestication some subtle sources of genomic diversification such as changes in the interactions among genes products and in the timing of gene expression may have influenced the diversity of the forms observed in the domestic dog [17, 70]. More deleterious mutations were detected in dog genome, compared with their wild ancestor. Our results similar to previous studies [15, 17] confirmed that domestication has increased deleterious mutations in domesticated animals than those in their wild ancestors.

It should be noted that mammalian genomes possess a complex structure with a diverseness of repetitive elements that complicates extensive genome-wide analyses [66]. To better acknowledge this result, there is still the need for using mate pair sequences or merging long-insert mate pair and short-insert paired-end sequences to analyze the dog and wolf genomes and elucidate the difference of the distribution and impact of the genomic variations between dog and wolf during dog domestication. More work using a larger sample size is needed to more clearly unravel genome changes during dog domestication and selective breeding.

## Conclusions

We resequenced the whole genomes of 6 canids from the Middle East for the first time and we compared the effect and distribution of the genomic variations between dog and wolf genomes. Whole genome resequencing of three dogs and three wolves detected 7.82 million and 10.45 million SNPs, respectively. Numerous putatively CNVs were identified through an analysis of read depth difference. Furthermore, we have identified SVs which could be useful for marker based population genetic investigation. Downstream analysis of the identified SVs and CNVs revealed the changes between dog and wolf genome during dog domestication.

## Methods

### Sampling and sequencing

The sources of the animals used in this study were as follow: one wolf was sampled from Kerman zoo, South of Iran, two wolves were used from Eram Park Zoo, Tehran, Iran; two Saluki dogs were sampled from private farms (Jamil Tavanaei) in Kurdistan province (Bijar and Sanandaj), west of Iran and one Qahderijani dog was used from a private farm (Alireza Hoseini) in Qahderijan**,** Falavarjan County, Isfahan Province, Iran. We collected blood samples from three captive Iranian wolves (Additional file 1: Fig. S17) and three Iranian dogs including a Qahderijani (Additional file 1: Fig. S18) and two Saluki dogs (Additional file 1: Fig. S19) with the consent of the owners. Sampling locations are reported in Table 3. DNA was prepared with phenol/chloroform technique. Pair-end sequence data for all 6 individuals were generated using Hiseq 2500 Illumina.

**Table 3:** Sampling location and ecotypes

Sample	Sample ID	Location	Ecotype	The latitude and longitude of each location
Dog	DogSI1	Sanandaj, Iran	Saluki (Tazi)	35 18′ 52″ N, 46 59′ 32″ E
Dog	DogSI2	Bijar, Iran	Saluki (Tazi)	35 52′ 22″ N, 47 36′ 10″ E
Dog	DogQI	Esfahan, Iran	Qahderijani	32 38′ 0″ N, 51° 39′ 0″ E
Wolf	GW1	Hamadan, Iran	–	34 48′ 0″ N, 48° 31′ 0″ E
Wolf	GW2	Tehran, Iran	–	35 41′ 46″ N, 51 25′ 23″ E
Wolf	GW3	Kerman, Iran	–	30 17′ 0″ N, 57 5′ 0″ E

### Quality control and mapping

The quality of the reads was evaluated with FastQC program and outputs of quality control showed that all reads had high-quality and were without adaptor contamination. Aligning data against the genome assembly canfam3.1 was done with Burrows-Wheeler Aligner program (BWA) [40]. The SAMtools [41] was applied to change the Sequence Alignment MAP (SAM) files to the Binary Alignment MAP (BAM) files and sort and index them. All of the .bam files were cleaned from PCR duplicates with Picard program. The accuracy of mapping was evaluated using of two criteria including percentage of aligning against the reference genome and mean depth with SAMtools.

### Short Indel and SNP detection

Genome Analysis Toolkit (GATK) program [46] was applied to detect SNPs and Indels. All .bam files were preprocessed in two steps; i) local realignment around Indels was done using known Indels, ii) recalibrating base quality scores was done to increase quality score for each base. The purified data belonged to the same individual were jointly used to create genome variant call format (gVCF) files by GATK HaplotypeCaller, followed by merging the gVCF files belonged to all individuals employing the GATK GenotypeGVCFs. Finally, SNPs and Indels were separated from the resulted raw variant file and filtered using GATK Select Variants and GATK Variant Filtration, respectively.

### SVs detection

SVs including deletions, inversions, translocations (inter and intra chromosomal) and insertions were detected by using both of BreakDancer-1.1 [18] and DELLY [58] software. SVs were filtered using BreakDancer with read coverage > = 10, the score > =80 and size> = 50 bp.

### SNP and Indel annotation

Functional consequence analysis of SNPs and short INDELs were predicted using SnpEff 4.0e [20]. The transition to transversion and homozygous to heterozygous ratios for single nucleotide variants were calculated with SnpSift [61].

### Prediction of deleterious mutations

To predict the deleterious mutations within all individual canid genomes, we used the SIFT (Sorting Intolerant from Tolerant) algorithm [50]. If this normalized value is less than 0.05, the substitution is predicted to be deleterious, and those greater than or equal to 0.05 are predicted to be tolerated.

### CNV calling

Putative CNVs on the 38 Canine autosomes and X chromosome were detected based on read depth method using CNVnator [1]. We run CNVnator with a bin size of 150 bp and GC correction (default) for our data. Filtering putative CNVs was done using different criteria including size > l kb, *P*-value < 0.01 and q0 (zero mapping quality) < 0.5. We removed all un-localized chromosome CNVs (chrUn). Putative CNVRs were obtained using Bedtools software [56] from overlapping of 1 bp or greater CNVs on chromosomes 1–38 and X chromosome in 6 individuals as suggested before [59]. All CNVRs were categorized into three classes, e.g., “Loss” (including deletion), “Gain” (including duplication) and “Both” (including both deletion and duplication). To compare the putative CNVRs from this study with the CNVRs reported in the previous studies, all coordinates related to CNVRs of the previous studies were converted from canFam2.0 to canFam3.1 using the lift over tools (https://genome.ucsc.edu/cgi-bin/hgLiftOver).

### Gene contents and gene ontology analysis

Dog gene IDs that covered small Indels, SVs and CNVRs were retrieved from Ensemble annotation [29]. All dog gene IDs were changed to human gene IDs. Gene orthologous connection between dog and human was obtained from Ensembl. Gene ontology (GO) was done using DAVID program [36].

### Visualization of structural genomic variation

We drew the physical distribution of CNVRs on chromosomes 1–38 and X chromosomes using vcstools [38]. RCircos package [79] was used to draw circular genetic maps for visualizing similarities and differences of positional relationships and genome structure between dog and wolf.

## Supplementary information

**Additional file 1: Tables S1**-**S15** and **Figs. S1-S19**.

**Additional file 2: Table S16.** Genomic structural variants including insertions, tandem duplication, deletions, translocations (inter and intra chromosomal) and inversions for three dogs.

**Additional file 3: Table S17.** Genomic structural variants including insertions, tandem duplication, deletions, translocations (inter and intra chromosomal) and inversions for three wolves.

**Additional file 4: Table S18.** The total number of deletions and inversions in dog and wolf genomes.

**Additional file 5: Table S19.** The total number of CNVRs.

**Additional file 6: Table S20.** Statistics of the detected CNVs for Canine autosomes and X chromosome.

**Additional file 7: Table S21.** Comparison with previous dog CNV studies.

## Data Availability

**Data deposition**: Raw sequence reads data have been deposited in the Genome Sequence Archive (http://gsa.big.ac.cn/) under accession CRA001324 for raw data of genomes.

## Abbreviations

3′-UTR: 3′ untranslated region
5′-UTR: 5’untranslated region
BAM: Binary Alignment MAP
BWA: Burrows-Wheeler Aligner program
CGH: Comparative genomic hybridization
chrUn: Un-localized chromosome
CNVRs: Copy number variation regions
CNVs: Copy number variations
FC: Fertile crescent
GATK: Genome Analysis Toolkit
GO: Gene ontology
gVCF: Genomic variant call format
GW: Gray wolf
Indels: Insertion and deletion
KEGG: Kyoto Encyclopedia of Genes and Genomes
QI: Qahderijani
SAM: Sequence Alignment MAP
SI: Saluki
SVs: Structural variants
