# Supplementary material for: Whole genome resequencing of the Iranian native dogs and wolves to unravel variome during dog domestication
Source: BMC Genomics. 2020 Mar 4;21:207. doi: 10.1186/s12864-020-6619-8 (PMC7057629; doi:10.1186/s12864-020-6619-8)
Supplement: Supplementary file 1 — Additional file 1: Tables S1-S15 and Figs. S1-S19. [file 12864_2020_6619_MOESM1_ESM.doc]

**Table S1.** Library size and throughput for different individuals

| Sample | ID | Location | Mean insert size | Reads Length | STD |
| --- | --- | --- | --- | --- | --- |
| Dog | DogSI1 | Sanandaj | 322.62  322.69 | 125  125 | 33.94  33.60 |
| Dog | DogSI2 | Bijar | 331.86  331.28  322.04 | 125  125  125 | 32.98  31.28  31.03 |
| Dog | DogQI | Esfahan | 310.91  310.88  310.36  310.51 | 125  125  125  125 | 28.49  29.07  28.46  28.80 |
| Wolf | GW1 | Hamadan | 280.41  280.06  280.38 | 125  125  125 | 30.12  29.91  29.38 |
| Wolf | GW2 | Tehran | 300.34  300.55  300.02 | 125  125  125 | 27.12  27.53  27.17 |
| Wolf | GW3 | Kerman | 297.76  298.54  298.88 | 125  125  125 | 27.58  28.57  28.23 |


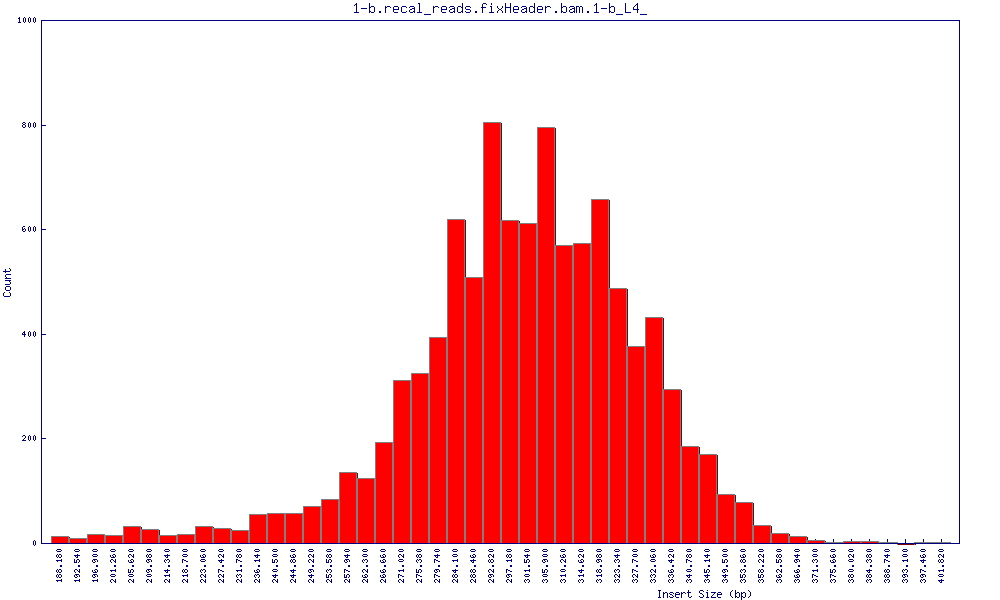


**Figure S1.** The distribution curve of insert sizes in on of. bam files.

**Table S2.** Sequencing output for the six samples.

| Total high quality  Sequence  (Gb) | Alignment Percent | Mean depth or coverage | Total number  of short reads aligned | Short reads length | Total number of short reads | ID Sample |
| --- | --- | --- | --- | --- | --- | --- |
| 46.5 | 99.58% | 15.02 | 299566646 | 125 | 300830756 | DogSI1 |
| 47.7 | 99.53% | 16.24 | 320224240 | 125 | 321722012 | DogSI2 |
| 51.0 | 98.45% | 16.82 | 326163070 | 125 | 327604955 | DogQI |
| 42.1 | 99.45% | 14.51 | 284280842 | 125 | 285864970 | GW1 |
| 50.9 | 99.25% | 17.15 | 332944929 | 125 | 334497849 | GW2 |
| 49.3 | 99.54% | 16.03 | 311172864 | 125 | 313534286 | GW3 |

**Table S3.** Number of single nucleotide polymorphisms and small insertions and deletions for 6 samples before and after filtering

| Sample | ID sample | raw or filtered | number of SNPs | number of Indels |
| --- | --- | --- | --- | --- |
| Dog | DogSI1 | Raw | 5060098 | 1285825 |
| Dog | DogSI1 | Filtered | 4658554 | 1282911 |
| Dog | DogSI2 | Raw | 5043139 | 1268977 |
| Dog | DogSI2 | Filtered | 4624244 | 1266491 |
| Dog | DogQI | Raw | 5144922 | 1347439 |
| Dog | DogQI | Filtered | 4741165 | 1344338 |
| Wolf | GW1 | Raw | 6876898 | 1776196 |
| Wolf | GW1 | Filtered | 6360953 | 1773198 |
| Wolf | GW2 | Raw | 6916727 | 1794569 |
| Wolf | GW2 | Filtered | 6430171 | 1790888 |
| Wolf | GW3 | Raw | 6921297 | 1772691 |
| Wolf | GW3 | Filtered | 6467804 | 1769908 |
| total raw |  | Raw | 13519228 | 3497597 |
| total filtered |  | Filtered | 12459651 | 3487342 |


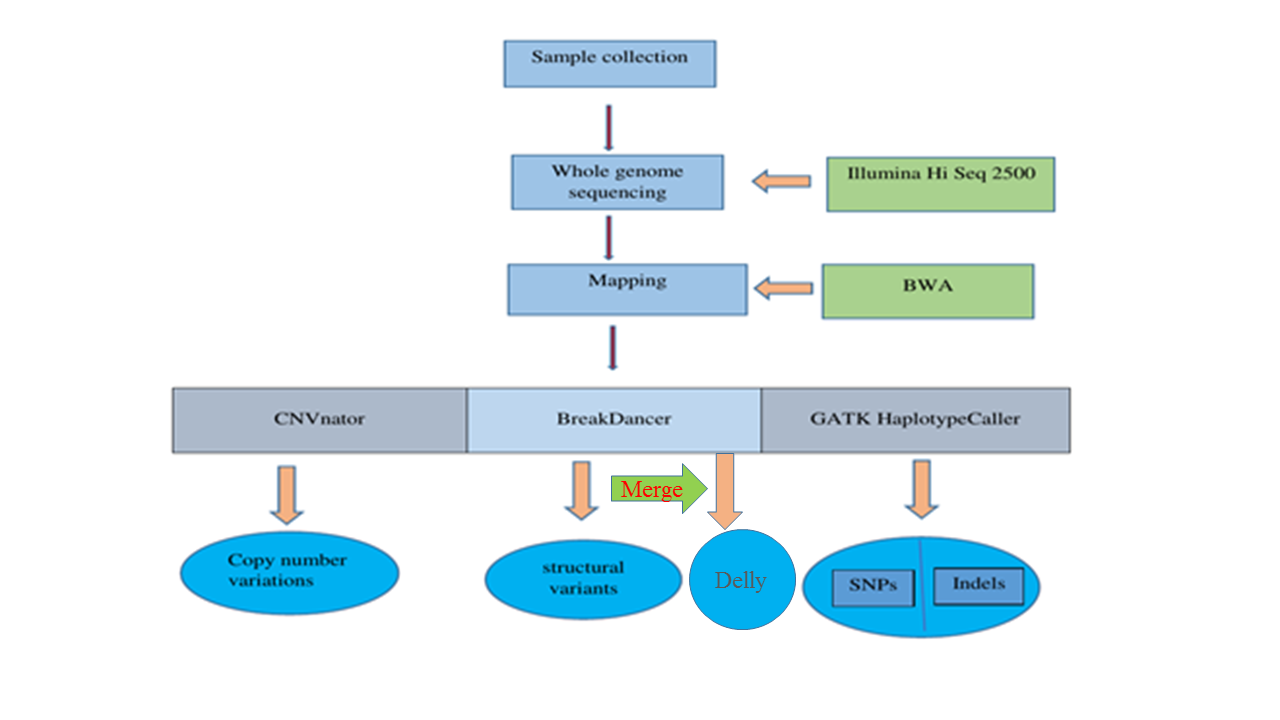


**Figure S2.** Workflow for our sequencing and analysis

**Table S4.** The number of transition, transversion and homozygous, hetrozygous

| sample | ID Sample | The number of Homozygous/ Heterozygous in SNP | The number of Homozygous / Heterozygous in indels | The number of transition in SNP | The number of transversons in SNP | Transition/ transversion ratio in SNP |
| --- | --- | --- | --- | --- | --- | --- |
| Dog | DogSI1 | 1873778/2784776 | 624450/658461 | 3108871 | 1549683 | 2.00 |
| Dog | DogSI2 | 1849780/2774464 | 606987/659504 | 3083331 | 1540913 | 2.00 |
| Dog | DogQI | 1768009/2973156 | 612488/731850 | 3156044 | 1585121 | 1.99 |
| wolf | GW1 | 2712589/3648364 | 872036/901162 | 4283743 | 2077210 | 2.06 |
| wolf | GW2 | 2534357/3895814 | 836374/954514 | 4328066 | 2102105 | 2.05 |
| wolf | GW3 | 2637476/3830328 | 850707/919201 | 4362918 | 2104886 | 2.07 |

**Table S5.** Number of SNP effects in different regions of genome

| Utr_5_  Prime | Utr_3_prime | Upstream | Transcript | Splice_  site_ region | Splice_  Site_ donor | Splice_ site_ acceptor | None | Intron | Inter  genic | Exon | Down  stream | Sample |
| --- | --- | --- | --- | --- | --- | --- | --- | --- | --- | --- | --- | --- |
| 12115  (0.13)  % | 40668  (0.46)  % | 446942  (4.89)  % | 62  (0.001)  % | 6722  (0.07)  % | 193  (0.002)  % | 243  (0.03)  % | 328247  (3.60)  % | 2909718  (31.81)  % | 4895571  (53.52)  % | 76811  (0.84)  % | 429701  (4.70)  % | Dog |
| 17714  (0.135)  % | 56747  (0.43)  % | 642810  (4.91)  % | 94  (0.001)  % | 9172  (0.07)  % | 289  (0.002)  % | 334  (0.003)  % | 421329  (3.22)  % | 4166631  (31.85)  % | 7053895  (53.92)  % | 105255  (0.81)  % | 607610  (4.65)  % | Wolf |
| 19332  (0.12)  % | 66712  (0.44)  % | 732293  (4.83)  % | 90  (0.001)  % | 10828  (0.07)  % | 282  (0.002)  % | 336  (0.002)  % | 533853  (3.52)% | 4849376(31.99)% | 8121058  (53.57)% | 122896  (0.81)  % | 702305  (4.63)% | Totall |

**Table S6.** Number of SNP effects by functional class

| Silent(Percent) | Nonsense(Percent) | Missense(Percent) | Sample |
| --- | --- | --- | --- |
| 42732(59.07%) | 290(0.40%) | 29309(40.52%) | Dog |
| 57712(58.33%) | 447(0.45%) | 40771(41.21%) | Wolf |
| 68899(59.55%) | 489(0.42%) | 46300(40.02%) | Total |

**Table S7.** Summary of small Indels in dog and wolf using GATk tools

|  |  | NO. of confident Indels |
| --- | --- | --- |
|  |  |  |
|  | Insertion | 1058701 |
| Dog | Deletion | 1184652 |
|  | Total indels | 2243353 |
|  | Insertion | 1428698 |
| Wolf | Deletion | 1680291 |
|  | Total indels | 3108990 |
|  | Insertion | 1583533 |
| Dog-wolf | Deletion | 1901311 |
|  | Total indels | 3484845 |


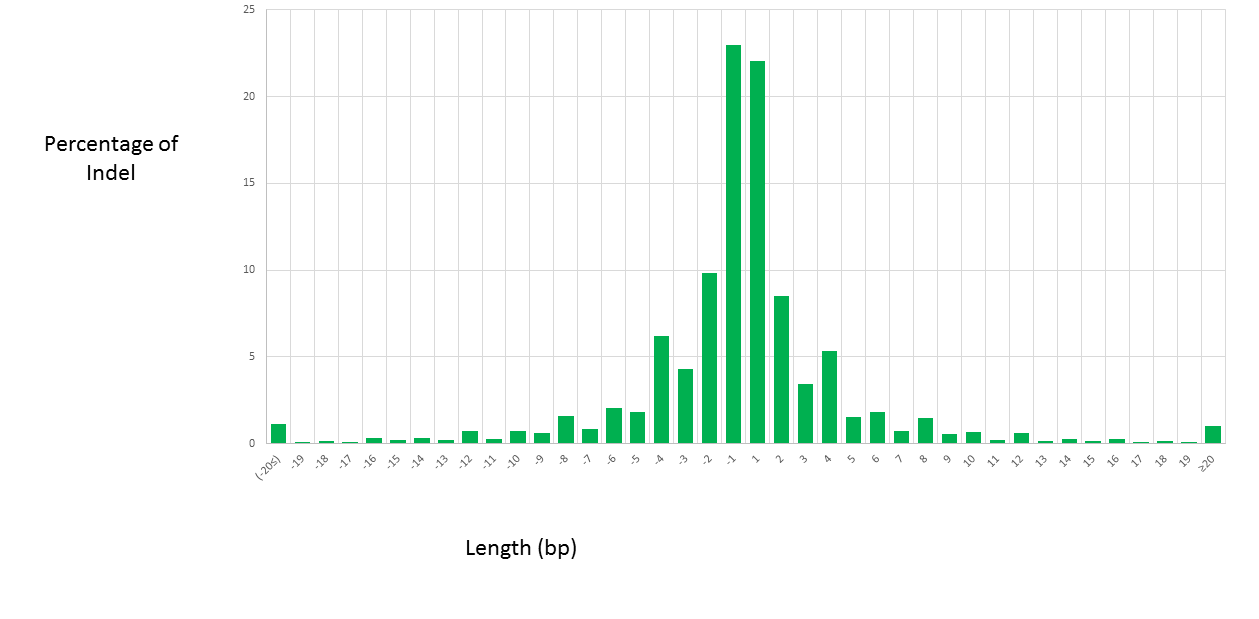
**Figure S3.** Indel length histogram across the 3 dog genomes. The positive and negative values are insertion and deletion, respectively.


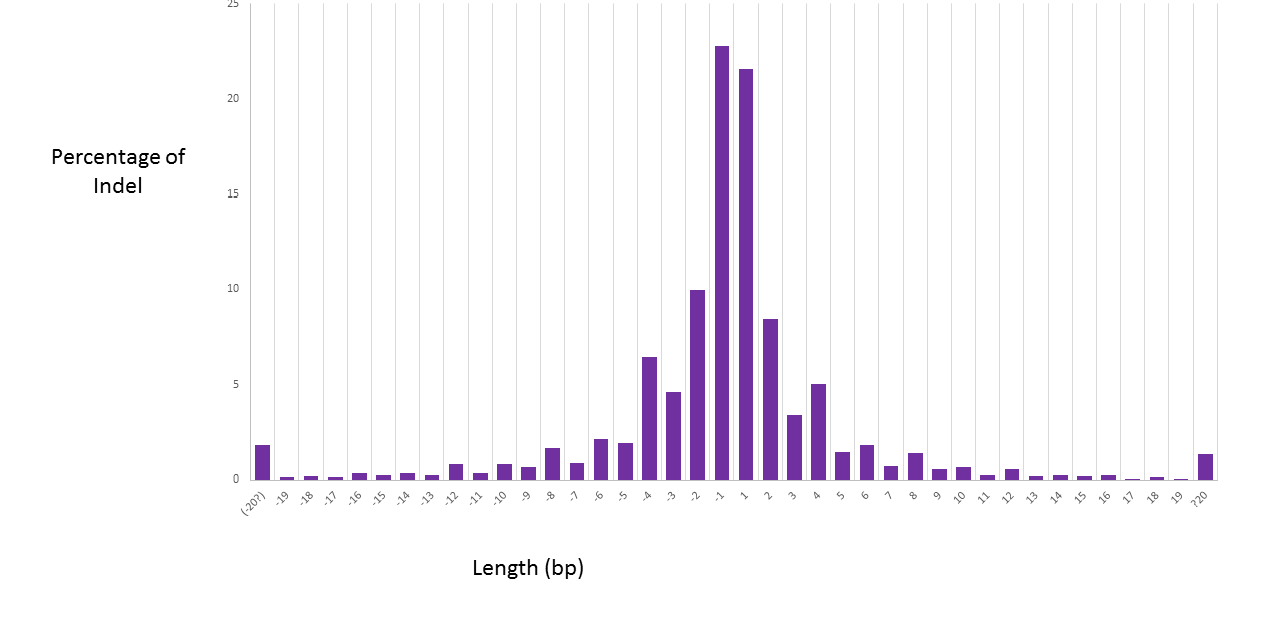


**Figure S4.** Indel length histogram across the 3 wolf genomes. The positive and negative values are insertion and deletion, respectively.


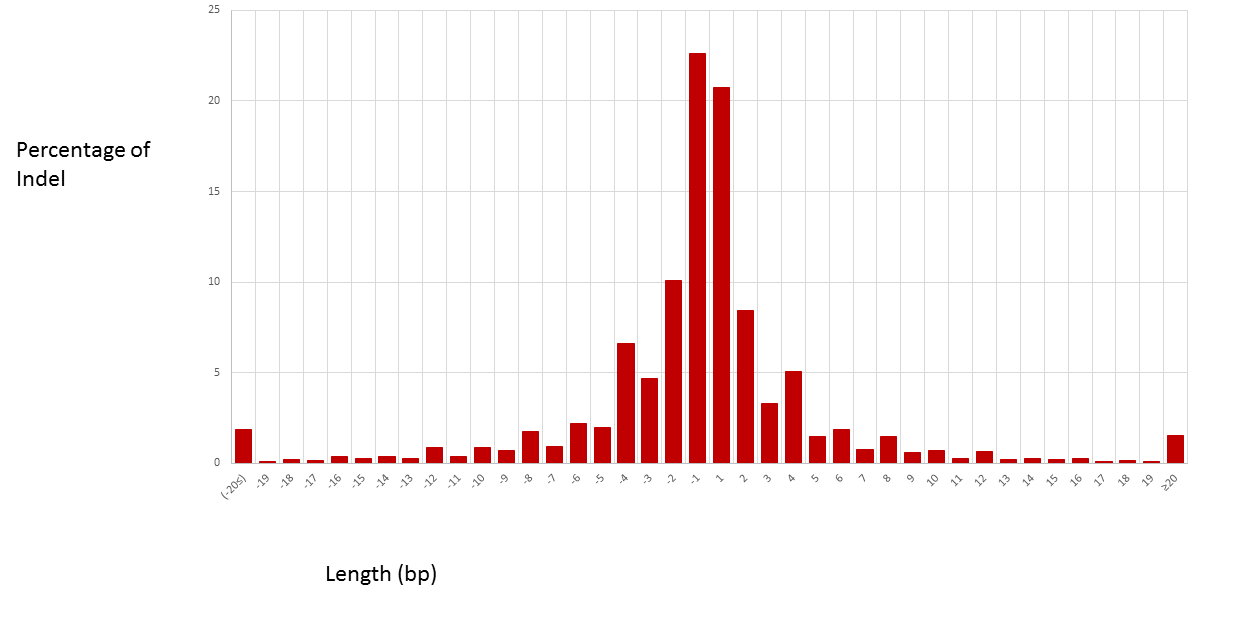


**Figure S5.** Indel length histogram across across six individual genomes. The positive and negative values are insertion and deletion respectively.

**Table S8.** Number of Indel effects in different regions of genome

| Utr_5_  prime | Utr_3_prime | Upstream | Transcript | Splice_  site_ region | Splice_  Site_ donor | Splice_ site_ acceptor | Gene | Intron | Inter  Genic | Exon | Down  Stream | Sample |
| --- | --- | --- | --- | --- | --- | --- | --- | --- | --- | --- | --- | --- |
| 3973  (0.15)  % | 13071  (0.48)  % | 154060  (5.62)  % | 61  (0.002)  % | 2034  (0.07)  % | 232  (0.01)  % | 370  (0.01)  % | 15  (0.001)  % | 951127  (34.69)  % | 1471275  (53.66)  % | 7483  (0.27)  % | 138369  (5.05)  % | Dog |
| 5483  (0.14)  % | 17552  (0.46)  % | 210772  (5.54)  % | 87  (0.004)  % | 2699  (0.07)  % | 230  (0.01)  % | 411  (0.01)  % | 29  (0.001)  % | 1321395  (34.75)  % | 2045818  (53.81)  % | 9214  (0.24)  % | 188446  (9.96)  % | Wolf |
| 5985  (0.14)  % | 19671  (0.46)  % | 235329  (5.54)  % | 103  (0.002)  % | 3064  (0.07)  % | 277  (0.01)  % | 596  (0.01)  % | 453  (0.01)  % | 1476727  (34.45)  % | 22832990 (53.79)% | 10407  (0.25)  % | 210059  (4.95)  % | Total |

**Table S9.** Gene Ontology terms enriched among Indels

| Ontology | GO ID | Description | Animal | P-wolf | P-dog |
| --- | --- | --- | --- | --- | --- |
| BP | GO:0007605 | sensory perception of sound | Both | 0.00596 | 0.004888 |
| BP | GO:0007601 | visual perception | Both | 0.013198 | 0.0105 |
| BP | GO:0006281 | DNA repair | Both | 0.001416 | 0.001042 |
| BP | GO:0006260 | DNA replication | Both | 0.010225 | 0.008326 |
| BP | GO:0045893 | positive regulation of transcription, DNA-templated | Both | 0.001085 | 0.001779 |
| BP | GO:0006366 | transcription from RNA polymerase II promoter | Both | 2.16E-06 | 3.94E-06 |
| BP | GO:0006338 | chromatin remodeling | Both | 0.008677  - | 0.007477 |
| BP | GO:0035725 | sodium ion transmembrane transport | Both | 0.002225 | 0.001909 |
| BP | GO:0098609 | cell-cell adhesion | Both | 7.53E-05 | 0.005164 |
| BP | GO:0006915 | apoptotic process | Both | 0.004557 | 0.002902 |
| BP | GO:0016568 | chromatin modification | Wolf | 0.046709 | - |
| BP | GO:0007010 | cytoskeleton organization | Dog | - | 0.047062 |
| BP | GO:0000398 | mRNA splicing, via spliceosome | Both | 0.038015 | 0.030925 |
| BP | GO:0016569 | covalent chromatin modification | Both | 0.039541 | 0.034365 |
| BP | GO:0043434 | response to peptide hormone | Both | 0.003173 | 0.002792 |
| BP | GO:0006811 | ion transport | Both | 0.00114 | 2.65E-04 |
| BP | GO:0006396 | RNA processing | Both | 5.94E-04 | 4.87E-04 |
| BP | GO:0000398 | mRNA splicing, via spliceosome | Both | 0.038015 | 0.030925 |
| BP | GO:0042787 | protein ubiquitination involved in ubiquitin-dependent protein catabolic process | Dog | - | 0.044134 |
| BP | GO:0006888 | Golgi vesicle-mediated transport | Dog | - | 0.049971 |
| MF | GO:0005524 | ATP binding | Both | 6.51E-07 | 1.65E-07 |
| MF | GO:0004842 | ubiquitin-protein transferase activity | Both | 0.031003 | 0.023407 |
| MF | GO:0008134 | transcription factor binding | Both | 0.04759 | 0.037387 |
| MF | GO:0005096 | GTPase activator activity | Both | 1.37E-04 | 8.99E-05 |
| MF | GO:0003725 | double-stranded RNA binding | Both | 0.036546 | 0.032761 |
| MF | GO:0003824~ | catalytic activity | Wolf | 0.030867 | - |
| MF | GO:0005249 | gated potassium channel activity | Wolf | 0.044096 | - |
| CC | GO:0030054 | cell junction | Both | 1.09E-07 | 1.30E-07 |
| CC | GO:0005730 | Nucleolus | Both | 6.60E-05 | 7.19E-05 |
| CC | GO:0005788 | endoplasmic reticulum lumen | Both | 0.001065 | 7.89E-04 |
| CC | GO:0016363 | nuclear matrix | Both | 0.00954 | 0.008043 |
| CC | GO:0005667 | transcription factor complex | Both | 0.005855 | 0.008703 |
| CC | GO:0043005 | neuron projection | Wolf | 0.046467 | - |
| CC | GO:0043235 | receptor complex | Wolf | 0.043251 | - |

**Table S10.** Summary of the structural variants in dog and wolf obtained using the Breakdancer software.

|  | Type | Software | NO. of confident SVs | Min.length  (bP) | Max.length  (bp) | Mean.length (bp) | Median.length (bp) |
| --- | --- | --- | --- | --- | --- | --- | --- |
| Dog | Insertion | BreakDancer | 352 | 125 | 247 | 168 | 169/943 |
|  | tandem duplication | Delly | 511 | 51 | 9017406 | 40423.19 | 271 |
|  | intra-chromosomal translocation | BreakDancer | 359 | 70 | 46214144 | 482561 | 856 |
|  | Deletion | Delly | 4886 | 51 | 15165644 | 212 | 9512.538 |
|  |  | Breakdancer | 10629 | 117 | 36886820 | 13205.337 | 219 |
|  |  | Merged | 13059 |  |  |  |  |
|  | Inversion | Delly | 290 | 60 | 19282816 | 166355.01 | 1087 |
|  |  | Breakdancer | 290 | 53 | 427377668 | 682558.8 | 28516.5 |
|  |  | Merged | 334 |  |  |  |  |
|  | inter-chromosomal translocation | Delly | 520 |  |  | .6 |  |
|  |  | BreakDancer | 434 |  |  |  |  |
| Wolf | Insertion | BreakDancer | 311 | 129 | 241 | 158.463 | 154 |
|  | tandem duplication | Delly | 666 | 63 | 23406476 | 52710.72 | 254.5 |
|  | intra-chromosomal translocation | BreakDancer | 421 | 63 | 46214200 | 451594.8 | 550 |
|  | Deletion | Delly | 6699 | 51 | 28317586 | 12213.55 | 208 |
|  |  | BreakDancer | 10661 | 107 | 9303672 | 5771.945645 | 217 |
|  |  | Merged | 18628 |  |  |  |  |
|  | Inversion | Delly | 388 | 54 | 45566988 | 761870.5 | 1147.5 |
|  |  | BreakDancer | 385 | 51 | 43664468 | 1019983 | 14386 |
|  |  | Merged | 401 |  |  |  |  |
|  | inter-chromosomal translocation | Delly | 706 |  |  |  |  |
|  |  | BreakDancer | 540 |  |  |  |  |

**Table S11.** The total number of genes that partially or completely have overlapped with different types of structural variants

| Sample | Type of Structural variant | The total number of genes that were retrieved from Ensemble | The number of Orthologous genes between the dog and human genome |
| --- | --- | --- | --- |
| Dog | Tandem Duplication | 839 | 582 |
|  | Indels (insertion and deletion) | 7812 | 5547 |
|  | Inversion | 6726 | 4547 |
|  | complex structural variants (inter and intra chromosomal translocations) | 224 | 220 |
| Wolf | Tandem Duplication | 1664 | 1139 |
|  | Indels (insertion and deletion) | 9782 | 6809 |
|  | Inversion | 5617 | 3661 |
|  | complex structural variants (inter and intra chromosomal translocations) | 254 | 228 |

**Table S12.** Gene Ontology terms enriched among the structural variants

| Ontology /type of variants | GO ID | Description | Animal | P-wolf | P-dog |
| --- | --- | --- | --- | --- | --- |
| BP/translocation | GO:0050911 | detection of chemical stimulus involved in sensory perception of smell | Both | 0.001377 | 9.27E-04 |
| BP/translocation | GO:0007608 | sensory perception of smell | Both | 0.00286 | 0.002248 |
| BP/translocation | GO:0007186 | G-protein coupled receptor signaling pathway | Both | 0.027544 | 0.039275 |
| BP/translocation | GO:0021775 | smoothened signaling pathway involved in ventral spinal cord interneuron specification | Both | 0.02621 | 0.024981 |
| BP/translocation | GO:0021776 | smoothened signaling pathway involved in spinal cord motor neuron cell fate specification | Both | 0.02621 | 0.024981 |
| BP/translocation | GO:0019323 | pentose catabolic process | Dog | - | 0.016724 |
| BP/translocation | GO:0009052 | pentose-phosphate shunt, non-oxidative branch | Dog | - | 0.049342 |
| BP/translocation | GO:0044262 | cellular carbohydrate metabolic process | Dog | - | 0.04129 |
| MF/translocation | GO:0004984 | olfactory receptor activity | Both | 0.001924 | 9.95E-04 |
| MF/translocation | GO:0004930 | G-protein coupled receptor activity | Both | 0.028142 | 0.006537 |
| MF/translocation | GO:0023024 | MHC class I protein complex binding | Both | 0.026946 | 0.024851 |
| MF/translocation | GO:1990405 | protein antigen binding | Both | 0.044508 | 0.041076 |
| MF/translocation | GO:0004750~ | ribulose-phosphate 3-epimerase activity | Dog | - | 0.016636 |
| MF/translocation | GO:0070330 | aromatase activity | Dog | - | 0.001441 |
| BP/ tandem duplication, | GO:0005975 | carbohydrate metabolic process | Dog |  | 0.06756 |
| BP/ tandem duplication, | GO:0021772 | olfactory bulb development | Both | 0.07 | 0.06 |
| BP/ tandem duplication, | GO:1904659 | glucose transmembrane transport | Dog | - | 0.07 |
| BP/ tandem duplication, | GO:0048666~ | neuron development | wolf | 0.06 | - |
| BP/ tandem duplication, | GO:0007417~ | central nervous system development | wolf | 0.004 |  |
| BP/ tandem duplication, | GO:0048169~ | regulation of long-term neuronal synaptic plasticity | wolf | 0.004 |  |
| BP/ tandem duplication, | GO:0007200~ | phospholipase C-activating G-protein coupled receptor signaling pathway | Both | 0.09 | 0.08 |
| BP/ tandem duplication, | GO:0035589~ | G-protein coupled purinergic nucleotide receptor signaling pathway | dog |  | 0.04 |
| CC/ tandem duplication, | GO:0006974~ | cellular response to DNA damage stimulus | dog |  | 0.07 |
| CC/ tandem duplication, | GO:0006975~ | DNA damage induced protein phosphorylation | wolf |  | 0.05 |
| CC/ tandem duplication, | GO:0030054~ | cell junction | Both | 0.008 | 0.002 |
| CC/ tandem duplication, | GO:0045202~ | synapse | wolf | 0.004 |  |
| MF/ tandem duplication, | GO:0045028~ | G-protein coupled purinergic nucleotide receptor activity | dog |  | 0.04 |
| MF/ tandem duplication, | GO:0005355~ | glucose transmembrane transporter activity | dog |  | 0.04 |
| BP/inversion | GO:0002682~ | regulation of immune system process | Both | 0.002616 | 0.006893 |
| BP/inversion | GO:0008643~ | carbohydrate transport | dog |  | 0.011505 |
| BP/inversion | GO:0015760~ | glucose-6-phosphate transport | dog |  | 0.03 |
| BP/inversion | GO:0042113~ | B cell activation | dog |  | 0.05 |
| BP/inversion | GO:0001913~ | T cell mediated cytotoxicity | dog |  | 0.05 |
| BP/inversion | GO:0007216~ | G-protein coupled glutamate receptor signaling pathway | dog |  | 0.05 |
| BP/inversion | GO:0050852~ | T cell receptor signaling pathway | Both | 0.012021 | 0.08 |
| BP/inversion | GO:0006111~ | regulation of gluconeogenesis | dog |  | 0.08 |
| BP/inversion | GO:0001580~ | detection of chemical stimulus involved in sensory perception of bitter taste | wolf | 1.79E-04 |  |
| BP/inversion | GO:0050909~ | sensory perception of taste | wolf | 0.003005 |  |
| BP/inversion | GO:0007399~ | nervous system development | wolf | 0.03 |  |
| CC/inversion | GO:0007155~ | cell adhesion | wolf | 0.01 | 0.03 |
| CC/inversion | GO:0030054~ | cell junction | Both | 0.082206 | 0.006042 |
| MF/inversion | GO:0003840~ | gamma-glutamyltransferase activity | Both | 0.011781 | 0.034656 |
| MF/inversion | GO:0033038~ | bitter taste receptor activity | wolf | 4.06E-06 |  |
| MF/inversion | GO:0023024~ | MHC class I protein complex binding | wolf | 0.065707 |  |
| MF/inversion | GO:0033038~ | bitter taste receptor activity | wolf | 4.06E-06 |  |
| BP/indels | GO:0006468~ | protein phosphorylation | Both | 6.69E-09 | 1.68E-09 |
| BP/indels | GO:0006468~ | protein phosphorylation | Both | 6.69E-09 | 1.68E-09 |
| BP/indels | GO:0007155 | ~cell adhesion | Both | 0.02 | 0.001522303 |
| BP/indels | GO:0007605~ | sensory perception of sound | Both | 0.017891 | 9.80E-04 |
| BP/indels | GO:0060996~ | dendritic spine development | Both | 0.025343 | 0.001270809 |
| CC/indels | GO:0005737~ | Cytoplasm | Both | 5.84E-14 | 6.78E-10 |
| CC/indels | GO:0005829~ | Cytosol | Both | 2.73E-18 | 6.34E-13 |
| CC/indels | GO:0016020~ | Membrane | Both | 7.24E-11 | 5.49E-09 |
| CC/indels | GO:0030054~ | cell junction | Both | 1.16E-09 | 1.48E-06 |
| CC/indels | GO:0005856~ | Cytoskeleton | Both | 2.65E-07 | 3.62E-07 |
| CC/indels | GO:0043197~ | dendritic spine | Both | 5.32E-07 | 4.04E-04 |
| CC/indels | GO:0030425~ | Dendrite | Both | 0.002799 | 4.72E-04 |
| CC/indels | GO:0043005~ | neuron projection | Both | 0.01287 | 1.20E-05 |
|  |  |  |  |  |  |
| CC/indels | GO:0030424~ | Axon | Both | 1.30E-04 | 1.31E-04 |
| CC/indels | GO:0005913~ | cell-cell adherens junction | Both | 0.001846 | 0.004244 |
| CC/indels | GO:0043235~ | receptor complex | Both | 4.24E-05 | 6.34E-04 |
| CC/indels | GO:0005654~ | Nucleoplasm | Both | 3.90E-04 | 0.031779 |
| MF/indels | GO:0044325~ | ion channel binding | Both | 0.001775 | 0.014176 |
| MF/indels | GO:0000287~ | magnesium ion binding | Both | 0.002451 | 0.019802 |
| MF/indels | GO:0005509~ | calcium ion binding | Both | 0.008068 | 0.015154 |
| MF/indels | GO:0005262~ | calcium channel activity | Both | 0.00475 | 0.026839 |
| MF/indels | GO:0019900~ | kinase binding | Both | 0.016348 | 0.086775 |
| MF/indels | GO:0004872~ | receptor activity | wolf | 0.02243 |  |
| MF/indels | GO:0003707~ | steroid hormone receptor activity | Both | 0.027934 | 0.034658 |
| MF/indels | GO:0051879~ | Hsp90 protein binding | Both | 0.054046 | 0.063374 |
| MF/indels | GO:0030544~ | Hsp70 protein binding | wolf | 0.067228 |  |


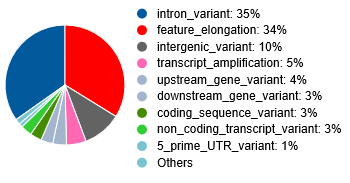

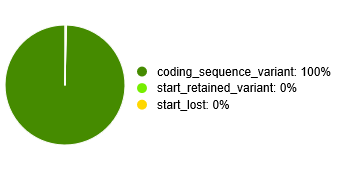


**Figure S6.** Annotation of results from indels in wolf genome.


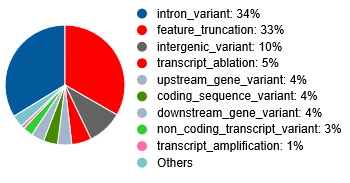

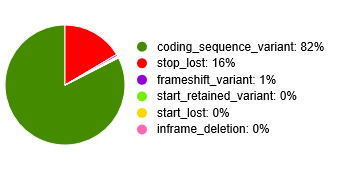


**Figure S7.** Annotation of results from indels in the dog genome.


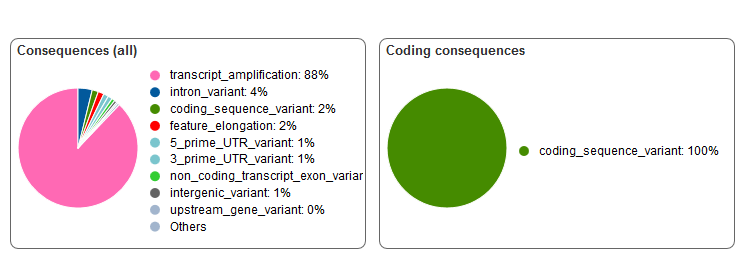


**Figure S8.** Annotation of results from inversions in the dog genome.


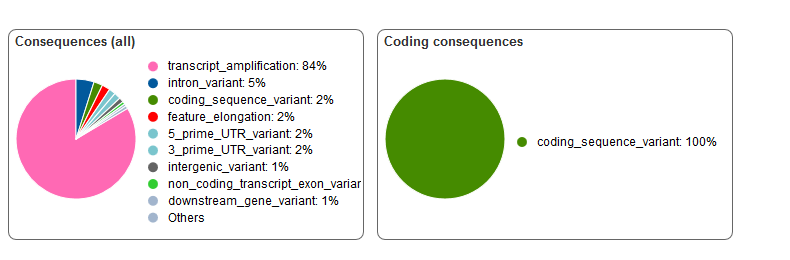


**Figure S9.** Annotation of results from inversions in the wolf genome.


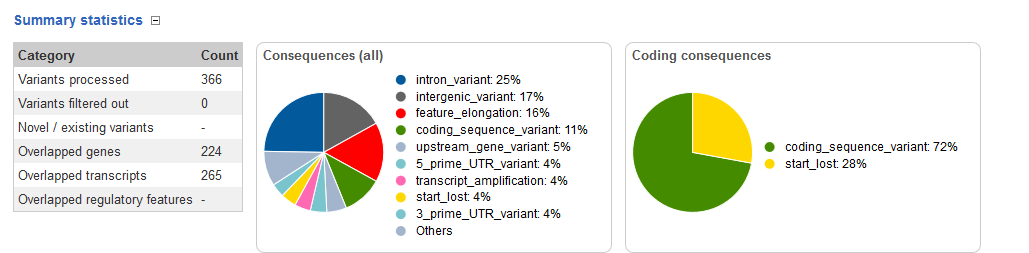


**Figure S10.** Annotation of results from translocations in the dog genome.


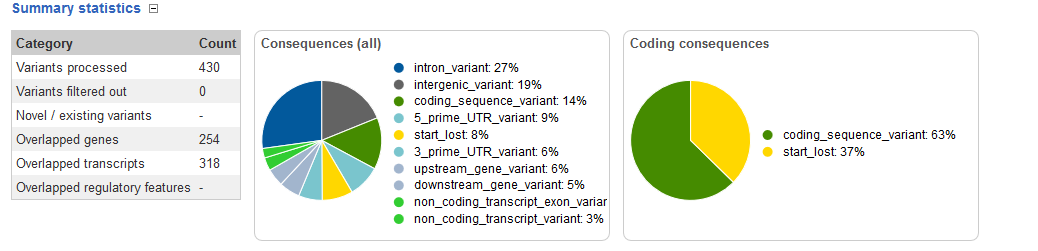


**Figure S11.** Annotation of results from translocations in the wolf genome.


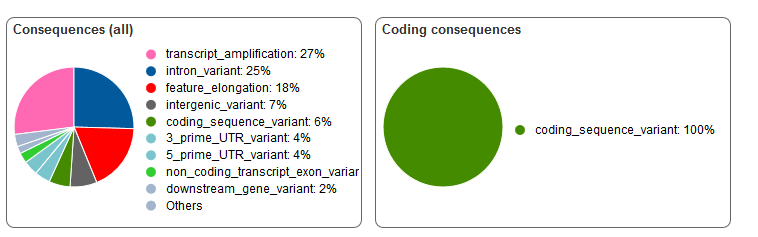


**Figure S12.** Annotation of results from tandem duplication in the dog genome.


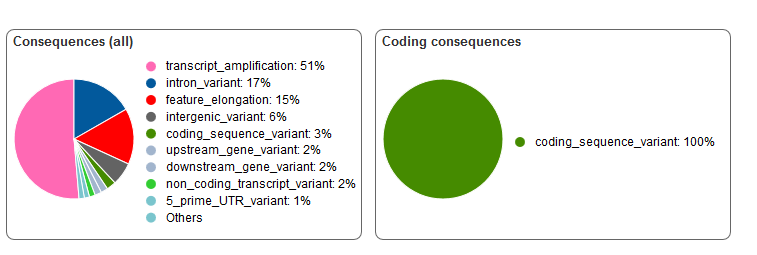


**Figure S13.** Annotation of results from tandem duplication in the wolf genome

**Table S13.** Number of CNVs in 6 samples

| Sample | ID | Total CNVs | Duplications | Deletions |
| --- | --- | --- | --- | --- |
| Dog | DogQI | 4683 | 1285 | 3398 |
| Dog | DogSI1 | 4010 | 2800 | 1210 |
| Dog | DogSI2 | 5437 | 2748 | 2689 |
| Wolf | GW3 | 3829 | 536 | 3293 |
| Wolf | GW2 | 4033 | 590 | 3443 |
| Wolf | GW1 | 2871 | 439 | 2432 |
| Mean |  | 4143.83 | 1399.66 | 2744.16 |

**Table S14.** Gene Ontology terms enriched among CNVs

| Ontology | GO ID | Description | Animal | P-wolf | P-dog |
| --- | --- | --- | --- | --- | --- |
| BP | GO:0007608 | sensory perception of smell | Both | 5.23E-13 | 2.92E-04 |
| BP | GO:0050907 | detection of chemical stimulus involved in sensory perception | Both | 1.95E-15 | 0.099396 |
| MF | GO:0004984 | olfactory receptor activity | Both | 2.55E-15 | 0.048229 |
| BP | GO:0019882 | antigen processing and presentation | Both | 0.006284 | 0.002908 |
| BP | GO:0007155 | cell adhesion | Both | 0.079404 | 0.032539 |
| BP | GO:0061337 | cardiac conduction | Dog | - | 0.03262 |
| MF | GO:0003823 | antigen binding | Both | 0.005492 | 0.097481 |
| MF | GO:0005506 | iron ion binding | Both | 0.010445 | 0.099795 |
| CC | GO:0005886 | plasma membrane | Both | 0.00234 | 3.90E-04 |
| CC | GO:0042611 | MHC protein complex | Both | 0.006417 | 0.046833 |
| MF | GO:0005516 | calmodulin binding | Both | 0.028426 | 0.095303 |
| MF | GO:0004930 | G-protein coupled receptor activity | Both | 2.86E-10 | 0.045 |
| MF | GO:0005096 | GTPase activator activity | Both | 0.002836 | 0.002611 |
| CC | GO:0042612 | MHC class I protein complex | Both | 1.49E-06 | 0.061726 |
| BP | GO:0008016 | regulation of heart contraction | Dog^*^ | - | 0.05729 |
| CC | GO:0005884~ | actin filament | Dog^*^ |  | 0.037 |
| BP | GO:0007507 | heart development | Dog^*^ |  | 0.080103 |
| BP | GO:0030049 | muscle filament sliding | Dog* |  | 0.025593 |
| BP | GO:0055001 | muscle cell development | Dog^*^ |  | 0.056071 |
| BP | GO:0048660 | regulation of smooth muscle cell proliferation | Dog^*^ |  | 0.080942 |
| MF | GO:0005524 | ATP binding | Dog^*^ |  | 3.46E-04 |
| MF | GO:0005509 | calcium ion binding | Dog^*^ |  | 0.001482 |
| MF | GO:0008307 |  | Dog^*^ |  | 0.013021 |

GO IDs related to the three GO categories (Molecular Function (MF), Cellular Component (CC), and Biological Process (BP)) enriched among the CNV gains in dog, wolf or both. P-values are presented for each of GO IDs in in the dog (P-dog) and in the wolf (P-wolf).

**Table S15.** Comparison between CNVRs detected in this study and previous studies

| Study | This study | Molin  *et al*.2014[48] | Berglund  *et al*.2012 [11] | Nicholas  *et al*.2011[51] | Axelsson  *et al*.2013[9] |
| --- | --- | --- | --- | --- | --- |
| Platform | Whole genome sequencing  (Illumina Hi Seq 2500) | CanineHD genotyping array | aCGH | aCGH | Whole genome sequencing  (ABSOLiD system, version3) |
| Total CNVR | 10571 | 72 | 394 | 616  [integrated with studies Chen *et al*. 2009 ; Nicholas *et al*. 2009] | 20813 |
| Reference genome | canFam3 | canFam2 | canFam2 | canFam2 | canFam2 |
| Successfully converted to canFam3^*^ | - | 70 | 347 | 533 | 17553 |
| Conversion failed | - | 2 | 47 | 83 | 3260 |
| Numbers of wolves | 3 | - | 3 | 1 [Nicholas *et al*.2009]  1 [Nicholas *et al.*2011]  - [chen *et.al*.2009] | 12 |
| Number of dog Breed | 2 | 30 | 17 | 17 [Nicholas *et al*.2009]  9 [Nicholas *et al*.2011]  7 [chen *et al*.2009] | 14 |
| Numbers of dogs | 3 | 395 | 50 | 17 [Nicholas *et al*.2009]  9 [Nicholas *et al*.2011]  9[chen *et al*.2009] | 60 |
| Overleaped CNVRs with this study | - | 136 | 250 | 440 | 4283 |

Note: All coordinates canfam2 were converted to canFam3 using Lift Over tool (<https://genome.ucsc.edu/>)


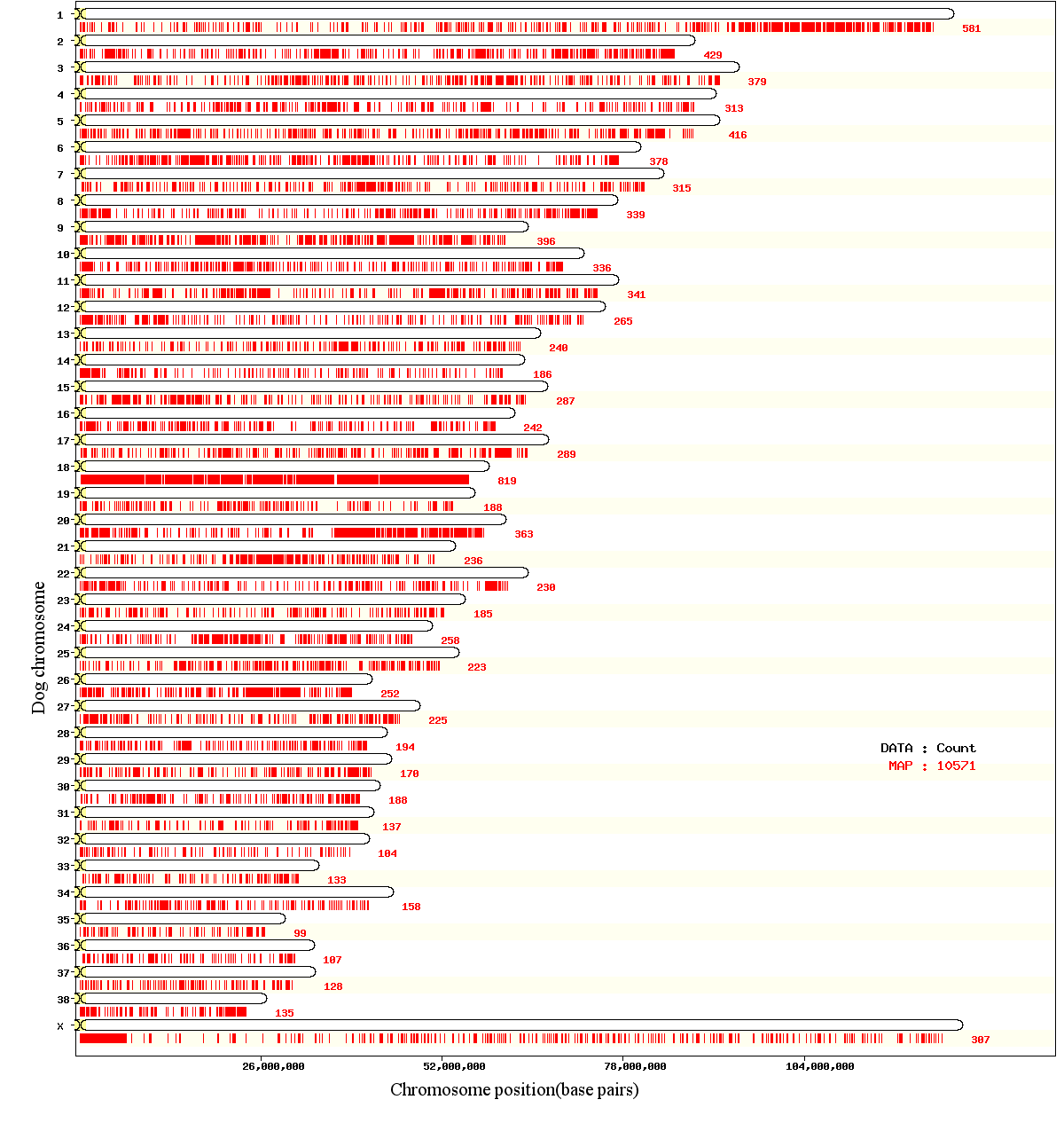


**Figure S14.** Distribution of CNVRs on chromosomes 1-38 and X chromosome


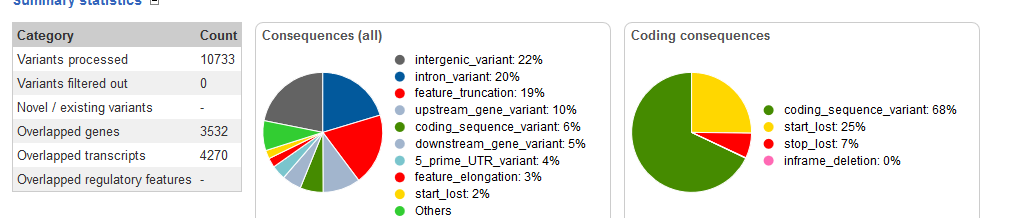


**Figure S15.** Annotation of results from CNVs in the wolf genome.


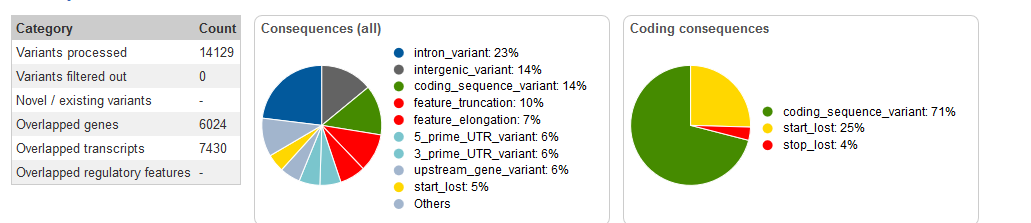


**Figure S16.** Annotation of results from CNVs in the dog genome.


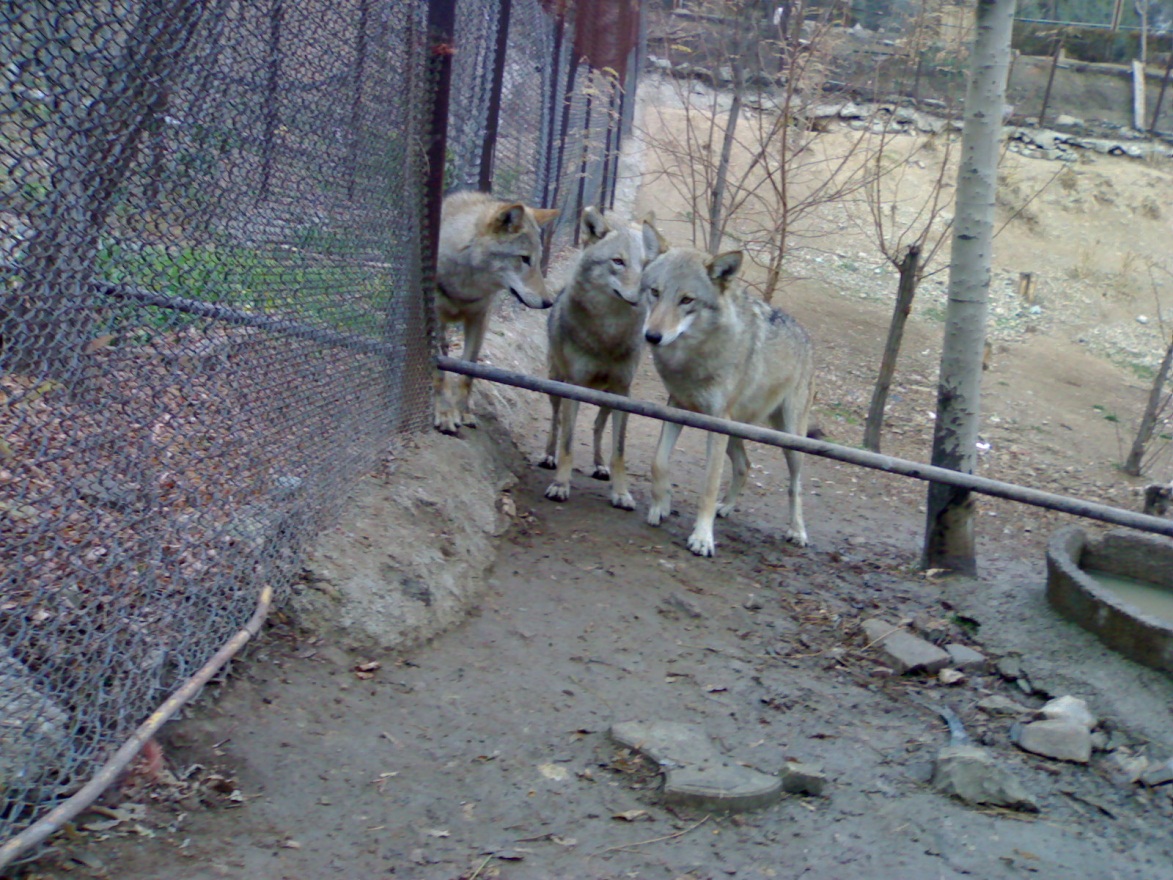


**Figure S17.** Iranian wolf (The photo was taken by Zeinab Amiri Ghanatsaman, the first author, from Eram Park Zoo, Tehran, Iran)


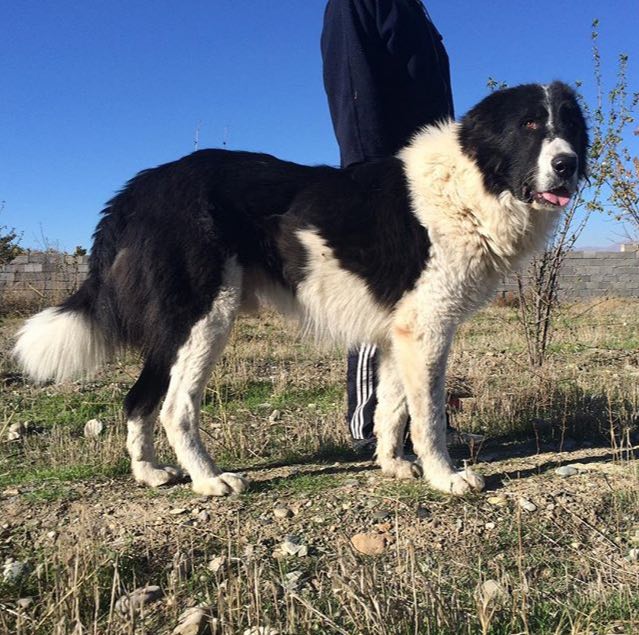


**Figure S18.** A Qahderijani dog (The photo was taken by Zeinab Amiri Ghanatsaman, the first author, from a private farm in Isfahan, Iran)


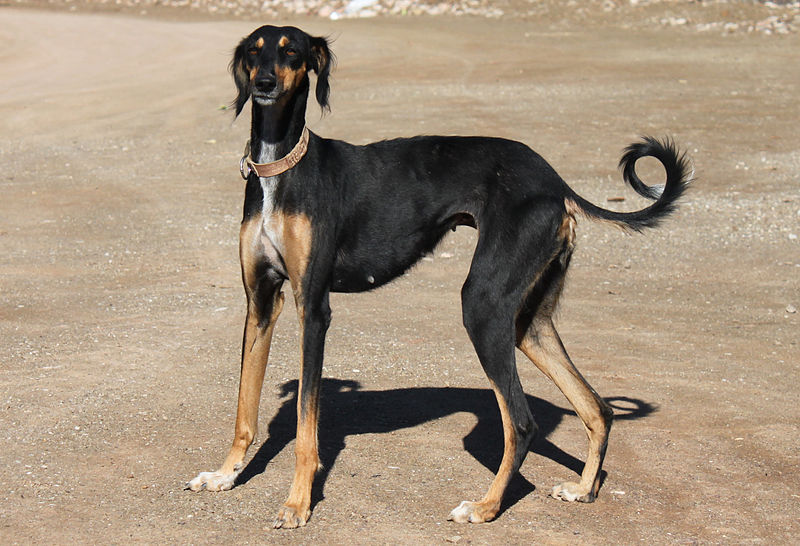


**Figure S19.** Persian saluki dog (https://commons.wikimedia.org/wiki/File:Persian_Saluki.JPG)
